# Direct evidence for metallic mercury causing photo-induced darkening of red cinnabar tempera paints

**DOI:** 10.1038/s42004-021-00610-2

**Published:** 2021-12-10

**Authors:** Kerstin Elert, Manuel Pérez Mendoza, Carolina Cardell

**Affiliations:** 1 Department of Mineralogy and Petrology, University of Granada, Avenida de Fuentenueva S/N, 18071 Granada, Spain; 2 Department of Inorganic Chemistry, University of Granada, Avenida de Fuentenueva S/N, 18071 Granada, Spain

**Keywords:** Photochemistry, Surface chemistry

## Abstract

Photo-induced darkening of red cinnabar (HgS) has attracted the interest of many researchers as it drastically impacts the visual perception of artworks. Darkening has commonly been related to metallic mercury (Hg^0^) formation in the presence of chlorides. Based on the study of UV-aged cinnabar pigment and tempera paint we propose an alternative pathway for the blackening reaction of cinnabar, considering its semiconductor properties and pigment-binder interactions. We demonstrate that darkening is caused by the oxidation of cinnabar to mercury sulfates and subsequent reduction to Hg^0^ via photo-induced electron transfer without the involvement of chlorides, and provide direct evidence for the presence of Hg^0^ on UV-aged tempera paint. Photooxidation also affects the organic binder, causing a competing depletion of photo-generated holes and consequently limiting but not impeding mercury sulfate formation and subsequent reduction to Hg^0^. In addition, organics provide active sites for Hg^0^ sorption, which is ultimately responsible for the darkening of cinnabar-based paint.

## Abstract (web summary)

Photo-induced darkening of red cinnabar (α-HgS) often dramatically changes the appearance of artworks, but the reduction mechanism of Hg^2+^ remains unclear. Here, the authors propose an alternative pathway for the blackening reaction of cinnabar, considering its semiconductor properties and pigment-binder interactions

## Introduction

**Fig. 1:**
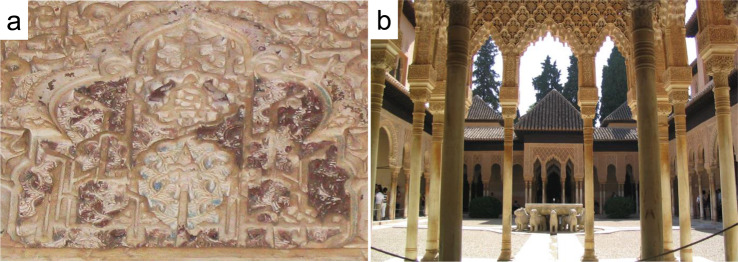
Altered cinnabar paint at the Alhambra palaces (Granada, Spain). **a** “Yesería” (plasterwork) with rests of altered cinnabar paint showing a dull dark-red color found on one of the capitals of the “Patio de los Leones” (**b**).

Photo-induced darkening of red cinnabar or synthetic vermilion (α-HgS) has been the topic of numerous studies as it often results in dramatic changes in the visual perception of artworks (i.e., mural, panel, or oil paintings), having affected important masterpieces such as Rubens’ Adoration of the Magi^1,2^ or polychrome Nasrid plasterwork at the Alhambra palaces (Granada, Spain) (Fig. 1). During the past few decades, researchers have studied historic paint samples in order to elucidate the cinnabar’s blackening process based on the analysis of alteration products^2–6^. Additionally, electrochemical experiments have been performed to further clarify the chemical changes undergone by cinnabar upon darkening, considering its semiconductor properties^1,7^. Cinnabar is an n-type semiconductor with a band gap energy of 2.0 eV and can be activated with visible light (<620 nm). Light with a wavelength >620 nm is reflected and responsible for the pigment’s intense red color.

Different pathways for the blackening reaction have been proposed, including the formation of black metacinnabar (β-HgS)^8,9^. Many researchers consider the formation of a thin layer of metallic mercury to be responsible for the darkening^1,2,6,10^, based on the fact that colloidal mercury is black^11^. However, direct evidence for its formation on altered cinnabar paint has not been provided so far^1,2,6,10^. Additionally, the imperative role played by chlorides has been recognized, either as a catalyst in the redox reaction of cinnabar or as intermediate reaction products that are subsequently photochemically reduced to metallic mercury^6,10^. Anaf et al.^1^ and Hogan and Da Pieve^12^ compared energy positions of the conduction and valence band edges of cinnabar (i.e., at pH 2 the valence and conduction band edges are situated at 2.02 and 0.02 V vs. NHE (normal hydrogen electrode), respectively, with the redox potential of cinnabar (i.e., HgS + 2e^−^ ↔ Hg^0^ + S^2−^ being equal to −0.70 V vs. NHE^13^). The authors concluded that, even though photooxidation might occur, a direct photo-induced reduction to metallic mercury was not possible because the redox potential was more negative than the conduction band energy, the energy of photo-excited electrons being insufficient to directly reduce Hg^2+^ to Hg^0^. Consequently, the authors proposed that the reduction to elemental mercury in cinnabar would only proceed in the presence of chlorides. This theory was justified by the fact that the redox potential of various mercury chloride compounds falls within the band gap of cinnabar (i.e., the potentials of HgCl_4_^2^^−^_(ads)_ + 2e^−^ ↔ Hg^0^_(ads)_ + 4Cl^−^ and Hg_2_Cl_2_ + 2e^−^ ↔ 2Hg^0^ + 2Cl^−^ are 0.48 and 0.27 V vs. NHE, respectively) and could, thus, be reduced to metallic mercury via photo-induced electron transfer^1,14^.

Several possible chloride sources have been considered: (a) impurities in the reactive ingredients used for the production of wet-process vermilion^15^; (b) protective coatings for wall paintings containing cera punica (beeswax boiled with seawater and niter)^5,16^; (c) sea spray in coastal areas^5^; (d) varnishes, binders, and consolidants used in painting conservation^17^; and (e) insecticides (organo-chlorides) and cleaning products (sodium hypochlorite) used for museum housekeeping^17^. Despite the fact that possible chloride sources are manifold, chlorides might not always be involved in cinnabar blackening^9^. Indeed, Cl-containing impurities or alteration products could not be detected in the case of naturally aged cinnabar-based paint mock-ups, even though they had undergone significant darkening upon 2-year outdoor exposure in the city center of Granada, southern Spain^18^. Daniels^15^ also reported on the darkening of vermilion (applied as a suspension on glass plates and exposed to daylight) in the absence of halides, which, however, only involved the outer edge of the sample and proceeded at a much slower rate as compared to samples containing NaCl.

In an effort to clarify whether cinnabar blackening can occur in the absence of chlorides and to elucidate the reactions involved in this process, natural cinnabar pigment and cinnabar-based tempera paint mock-ups were exposed to ultraviolet (UV) aging at room *T* and high relative humidity to create a highly oxidative environment. Actually, high humidity has been identified as an important factor during photocorrosion^10,19,20^. The pigments and egg yolk-based tempera paints used in our study were analyzed with multiple techniques to determine textural and compositional changes upon aging and to ascertain that they did not contain any chlorides. Based on our experimental results, an alternative pathway for the blackening reaction in the absence of chlorides is proposed that takes the semiconductor properties of cinnabar and pigment–binder interactions into account.

## Results

### Characterization of cinnabar pigment and tempera paint

The X-ray diffraction (XRD) pattern of the Chinese cinnabar pigment used in this study was in very good agreement with a natural reference cinnabar (International Centre for Diffraction Data (ICDD) file number: 897103). Field emission scanning electron microscopy–energy dispersive X-ray spectroscopy (FESEM-EDS) allowed the identification of various impurities. In backscattering imaging mode, a very small number of crystals containing Si, Fe, Zn, Ca, Mg, and Ba were detected^18^. Their elemental composition is compatible with the following minerals: quartz (SiO_2_), pyrite/marcasite (FeS_2_), zinc blende (sphalerite, ZnS), calcite/dolomite (CaCO_3_/CaMg(CO_3_)_2_), and barite (BaSO_4_). All these minerals are commonly associated with cinnabar ores^21^. The fact that these phases, with the exception of quartz and barite, were not detected by XRD indicates that they were present in concentrations below the detection limit of this technique (i.e., 2–3 wt%). Micro X-ray fluorescence (μ-XRF) results (Supplementary Table 1) showed some variations in Mg, Ca, and Fe concentration, pointing to an inhomogeneous distribution of accessory mineral grains. Additionally, P, Na, and K were detected in the cinnabar paint, all elements originating from the egg yolk binder. Importantly, Cl was neither detected in the pigment nor in the paint mock-ups with the aforementioned analytical techniques.

### Alteration of cinnabar pigment

**Fig. 2:**
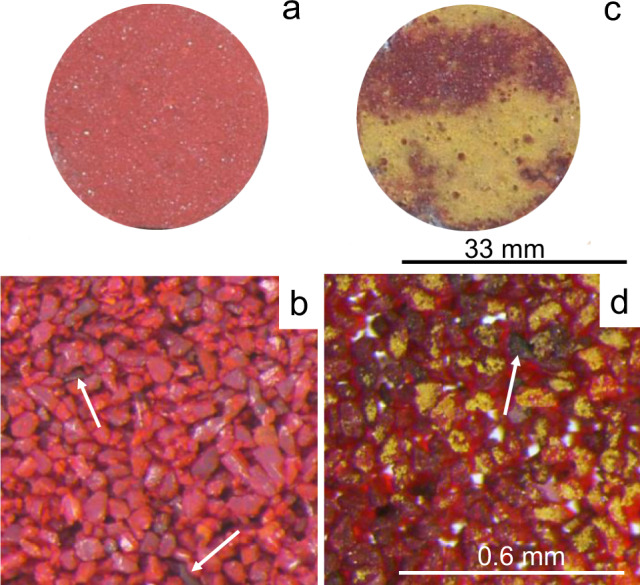
Chromatic and textural features of cinnabar pigment. **a**, **b** Unaltered (control) sample (scale bars as in **c**, **d**, respectively) and **c**, **d** 2 months after UV aging. Arrows indicate dark-colored grains corresponding to accessory minerals.

Photographic and optical microscopic images (Fig. 2) revealed darkening and the formation of a dense yellow layer, covering large parts of the UV-aged pigment surface after two months. Images of the unaltered pigment (control) are included for comparison. In addition, very few dark grains were observed in both samples (arrows), which correspond to the aforementioned accessory minerals, but did not have an important impact on the color of the bulk material.

**Fig. 3:**
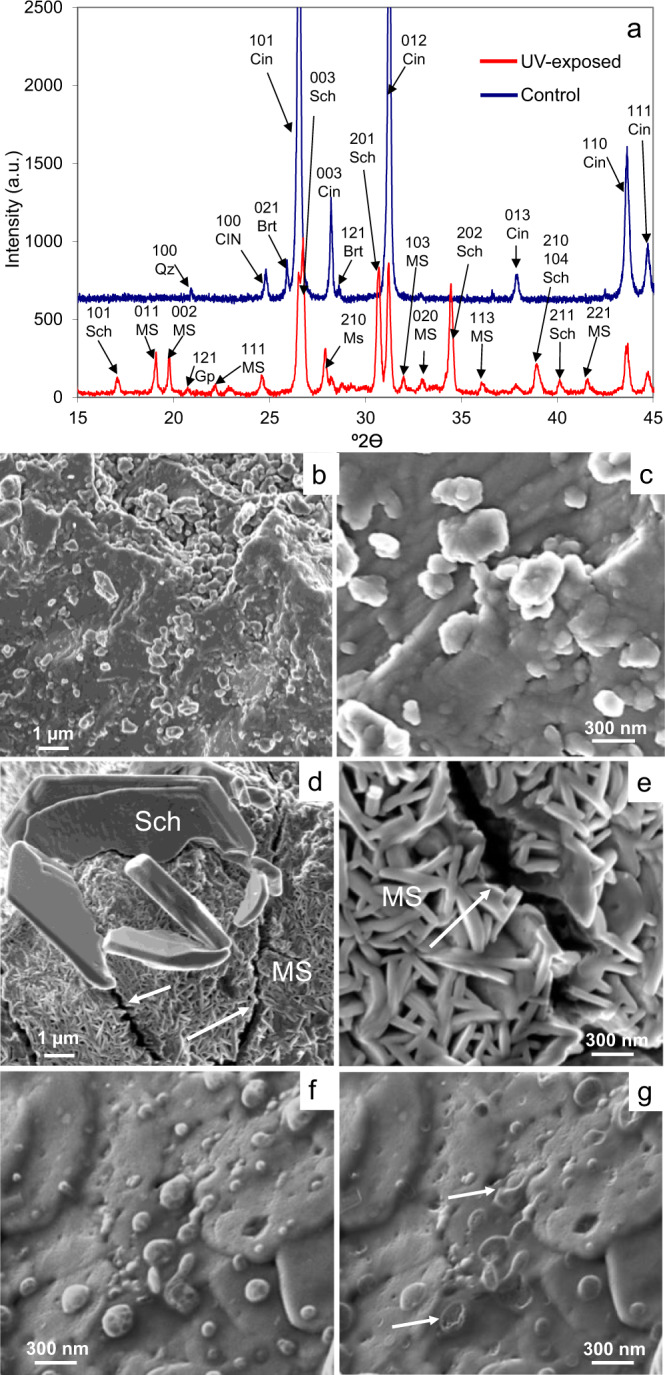
XRD patterns and FESEM images of unaltered and UV-exposed cinnabar pigment. **a** XRD patterns of unaltered (control) and UV-exposed cinnabar pigment after 2-month aging. Sch schuetteite, MS mercury sulfate hydrate, Cin cinnabar, Qz quartz, Gp gypsum, Brt barite (mineral abbreviations, except schuetteite and mercury sulfate hydrate, according to Whitney and Evans^70^; **b**, **c** FESEM images of the unaltered pigment showing a rough surface and nano-sized particles; **d** detail of the UV-exposed pigment surface showing cracks (arrows) and relatively large plate-like crystals (5–10 μm in size) corresponding to schuetteite; **e** close-up view of the UV-exposed pigment surface showing cracks (arrow) and small plate-like crystals (mercury sulfate hydrate), which form star-shaped structures and cover the entire pigment grain surface; **f**, **g** droplets (presumably mercury) on UV-exposed pigment surface before and after electron beam impact causing evaporation (arrows).

XRD analysis of the UV-exposed sample revealed the formation of mercury sulfate hydrate (HgSO_4_·H_2_O, ICDD file no: 742314) and basic mercury(II) sulfate (schuetteite^19^, Hg_3_(SO_4_)O_2_, ICDD file no: 120724) after 4 weeks (Fig. 3a). Mineralogical changes were accompanied by a ~50% reduction in the Bragg peak intensity of cinnabar, indicating extensive pigment degradation after 2 months of UV aging. In addition, a small amount of gypsum (CaSO_4_·2H_2_O) was detected, which was likely the product of a sulfation process undergone by calcite impurities in direct contact with the decomposing HgS^5^.

Furthermore, broadening of cinnabar Bragg peaks occurred, implying a decrease in crystallite size (i.e., average crystallite size reduction was 9 ± 2%, Supplementary Table 2). Broadening was angle dependent, indicating that it was likely strain-related^22^. According to Martinelli^23^ intergrowth of secondary phases can be the cause of stress, leading to microstrain-like peak broadening. In this respect, the formation of mercury sulfates and the accompanying volume increase have to be considered (i.e., the molar volume of HgS, Hg_3_(SO_4_)O_2_, and HgSO_4_·H_2_O being 28.7, 89.2, and 58.0 cm^3^/mol, respectively, and the corresponding theoretical volume increase upon transformation of HgS to Hg_3_(SO_4_)O_2_ and HgSO_4_·H_2_O being 3.5 and 102%, respectively). The XRD pattern of the unaltered pigment (control) is included for comparison (Fig. 3a), showing low-intensity Bragg peaks of quartz and barite additional to cinnabar. Finally, XRD did not allow the detection of either metacinnabar or chloride-bearing phases in any of the pigment samples.

FESEM results confirmed that the cinnabar pigment surface underwent drastic changes upon UV exposure. For comparison, FESEM images of the unaltered pigment (control) are included, showing a rough surface and some scattered nano-sized grains (Fig. 3b, c and Supplementary Fig. 1a). Photo-induced alteration was characterized by crack formation (Fig. 3d, e and Supplementary Fig. 1b) and the appearance of a new phase comprised of aggregated small plates (500 nm in size) forming star-shaped crystals, which covered the entire pigment surface. Having an Hg/S atomic ratio of 1 according to FESEM-EDS, this phase most likely corresponds to the mercury sulfate hydrate identified by XRD, which formed as a result of fluid-assisted mineral replacement via dissolution-precipitation^24^. In addition, abundant larger plate-like crystals (5–10 µm in size) were detected covering the star-shaped crystal layer of numerous pigment particles that displayed a yellow surface layer according to optical microscopy (Fig. 3d and Supplementary Fig. 1c, d). This latter phase had an Hg/S atomic ratio of 1.5 according to FESEM-EDS and likely corresponds to schuetteite, which according to Bailey et al.^19^ is canary yellow and forms superficial layers of small tabular hexagonal crystals on sunlight-exposed cinnabar. Both new phases only contained Hg and S according to FESEM-EDS. The aforementioned volume increase upon mercury sulfate formation can certainly be considered responsible for the observed crack development, leading to an increase in reactive surface area and enabling weathering at greater depth and faster rate. Reaction-induced fracturing has been reported for several mineral replacement reactions accompanied by volume increase (e.g., serpentinization of olivine)^25^. In addition, some nano-sized droplets were detected on the surface of a few of the UV-exposed pigment grains. These droplets suffered extensive electron beam damage, causing evaporation during analysis (Fig. 3f, g). Presumably, they correspond to metallic mercury, even though an unambiguous determination of their chemically composition was not possible due to the limited droplet size, EDS spectra only revealing the presence of Hg and S. Importantly, chloride was not detected in any of the samples.

**Fig. 4:**
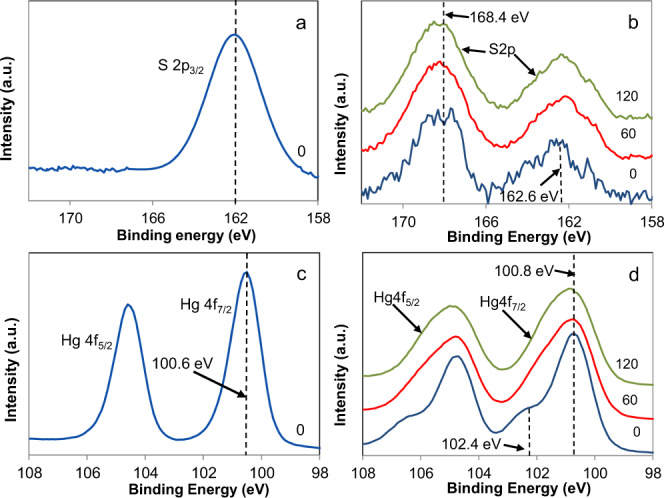
XPS spectra of unaltered and UV-exposed cinnabar pigment. **a**, **c** XPS spectra showing spectra of S 2p_3/2_ and Hg 4f_5/2_ and Hg 4f_7/2_ in unaltered cinnabar pigment, and **b**, **d** UV-aged pigment upon ion beam etching (etch time in seconds).

X-ray photoelectron spectroscopy (XPS) data of the UV-exposed cinnabar pigment revealed that the oxidation states of Hg and S had experienced important photo-induced changes. For comparison, the XPS spectrum of unaltered cinnabar pigment is included (Fig. 4a), which only shows a single component centered at 162 eV (i.e., the reported binding energy of the S 2p_3/2_ component in cinnabar is 161.2–162.2 eV)^26^. After 2 months, a signal corresponding to sulfates at 168.4 eV was detected (i.e., the reported binding energy of the S 2p_3/2_ component in mercury sulfates being 168.5–169 eV)^26^, which amounted to ~60 at% of the total S and remained unchanged upon Ar etching (Fig. 4b). The binding energy of the remaining S 2p_3/2_ component was centered at ~162.6 eV and did not change significantly upon Ar etching. A signal at 162.5–164.5 eV is commonly associated with the presence of elemental S, polysulfides or a metal-deficient sulfide layer^27^. Considering that surface sulfide ions (S^2−^) are more likely oxidized to sulfate (SO_4_^2−^) than to sulfur (S^0^) in the presence of oxygen^28^, and that schuetteite, having a Hg/S molar ratio of 3/1, will take up important amounts of Hg^2+^, it seems not unreasonable to assign this signal to a metal-deficient sulfide layer.

The Hg 4f_7/2_ band of unaltered pigment was centered at 100.6 eV (Fig. 4c), value close to that typically reported for cinnabar (i.e., 100.8–101.0 eV)^26^. In the case of the UV-aged pigment, the Hg 4f_7/2_ band included two components (Fig. 4d). One was centered at 100.8 eV, mounting to 75–80 at% of the total Hg. Note that it is difficult to differentiate cinnabar and mercury sulfate phases based on the Hg 4f_7/2_ signal, because the oxidation state of Hg is identical in both phases and the reported binding energy values are very similar. A second component was initially at 102.4 eV, but shifted to slightly lower values (i.e., 102.0 eV) upon Ar etching. Presumably, this component consisted of an oxidized Hg species. However, due to the lack of reference data it was not possible to associate it with any particular Hg compound^26^. Ehrhardt et al.^29^ observed a similar phenomenon and ascribed it to the presence of Hg(II), sorbed onto partially oxidized pyrite, Hg(II) having a high affinity for sulfur compounds. They concluded that this shift was not simply due to a change in the mercury’s oxidation state but rather related to a charge effect generated by the partially oxidized pyrite surface (i.e., FeS_2_ oxidized to Fe(III)oxyhydroxide). In the case of the UV-aged cinnabar pigment in this study, the oxidized sulfide surface might have exerted a similar charge effect on adsorbed Hg(II) as the oxidized pyrite in the cited study^29^. The presence of Hg^0^ (i.e., 99.7–99.9 eV)^26^ could not be confirmed with XPS. It cannot be ruled out that prolonged exposure to ultra-high vacuum conditions during analysis caused evaporation of loosely bond Hg^0^. Importantly, no Cl was detected in any of the analyzed samples.

### Alteration of cinnabar-based tempera paint

**Fig. 5:**
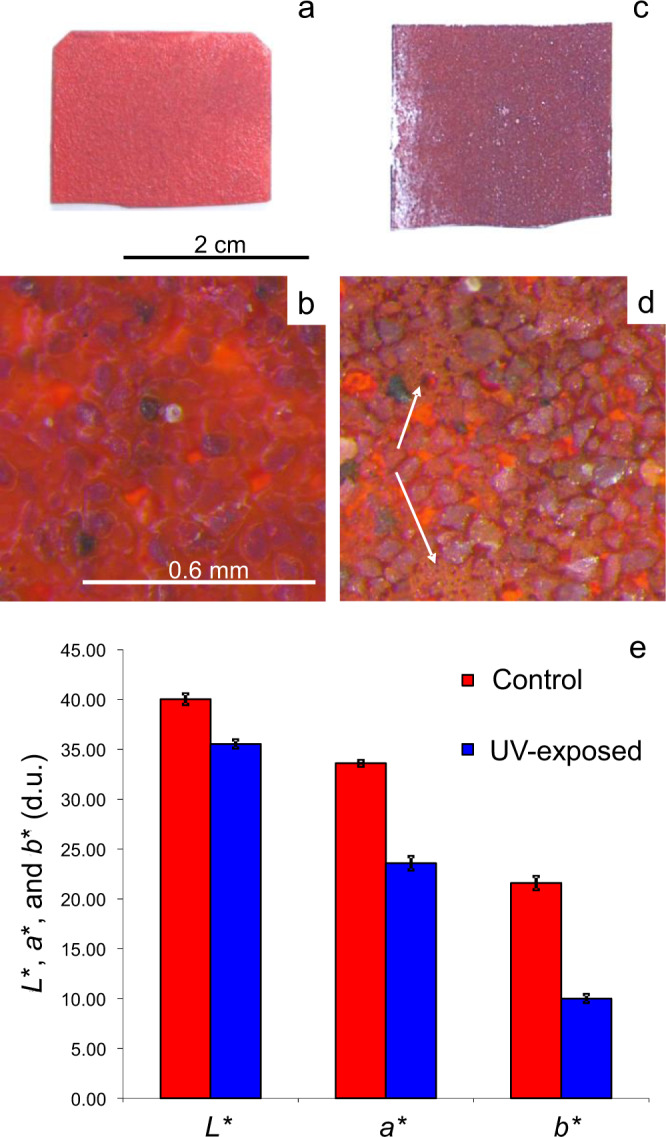
Photographic and microscopic images of unaltered and UV-exposed paint mock-ups. **a**, **b** Unaltered control and **c**, **d** paint sample after 2 months of UV exposure (arrows indicate degraded binder, scale bars as in **a**, **b**, respectively); **e** colorimetric coordinates (error bars show standard deviation) of unaltered (control) and UV-aged paint samples.

The tempera paint mock-up exposed to UV radiation suffered important darkening, but a yellow coating due to schuetteite formation as in the case of the cinnabar pigment was not detected (Fig. 5). Images of the unaltered control are included for comparison (Fig. 5a, b). Microscopic images showed an important degradation of egg yolk, leading to substantial binder loss which left pigment grains largely uncovered after prolonged UV exposure. However, some degraded binder remained in void spaces between pigment particles (Fig. 5d, arrows). XRD neither showed a reduction in peak intensity or peak broadening, nor the presence of Hg sulfate phases in the case of UV-exposed paint (Supplementary Fig. 2), but gypsum and possibly goethite (FeO(OH)) and zinc sulfate (ZnSO_4_) were identified, indicating that accessory minerals such as calcite, pyrite, and sphalerite in direct contact with the decomposing cinnabar suffered sulfation/oxidation during UV aging^30–32^.

Color measurements confirmed visual and microscopic observations, revealing a severe photo-induced color change (i.e., total color difference (ΔE*) = 16.0 ± 0.7; see methods section for details on color change calculation) due to an important decrease in L*, a*, and b*, indicating a change toward a darker, bluish-greenish color (Fig. 5e).

**Fig. 6:**
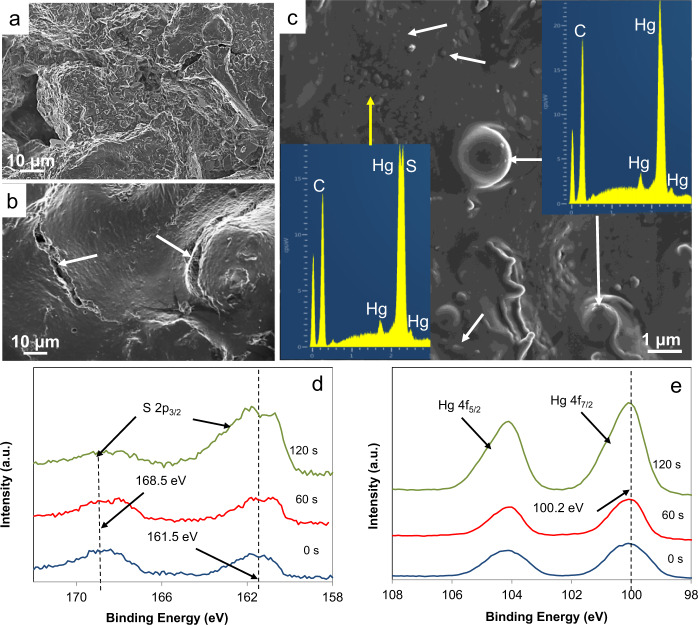
FESEM images and XPS spectra of paint mock-ups. **a** UV-exposed paint mock-up showing severe binder loss; **b** unaltered paint mock-up showing drying cracks (arrows) and **c** nano- to micrometer-sized mercury droplets (ranging from <100 nm to 1.5 µm, white arrows) on the mock-up surface after UV exposure (insets show EDS spectra of Hg^0^ droplet (white arrows) and HgS substrate (yellow arrow)); **d**, **e** depth profiles of S 2p_3/2_ and Hg 4f_5/2_/Hg 4f_7/2_ upon ion beam etching (etch time in seconds) of cinnabar paint exposed to UV radiation for 2 months.

FESEM images confirmed the important degradation of the egg yolk binder upon UV exposure (Fig. 6a). For comparison, an image of the unaltered paint sample (control) is included, showing some drying cracks in the organic binder (arrows, Fig. 6b). Remarkably, nano- and micrometer-sized mercury droplets (ranging from <100 nm to 1.5 µm) were observed on the UV-exposed mock-up (Fig. 6c), which withstood the high 10^−6^ Pa vacuum and electron beam impact during FESEM analysis. Likely, smaller mercury nanodroplets (similar to those observed in the FESEM image) coalesced and formed these micrometer-sized droplets in order to minimize surface energy. Mercury has a high surface tension (i.e., 486.5 mN/m) and the contact angle on many mineral surfaces^33^ including polished cinnabar is ~140° (Supplementary Fig. 3). Remarkably, the contact angle on the UV-exposed cinnabar paint surface observed in FESEM images was substantially lower (~105°). Considering the Young’s equation, this suggests a reduction in interfacial energy between the substrate and the liquid mercury, likely due to bonding via oxygen functional groups of the degraded organic binder (see “Discussion”).

XPS spectra of the UV-exposed cinnabar paint mock-up revealed the presence of sulfates. The ratio of the two S 2p_3/2_ signals centered at ~161.5 and ~168.5 eV (Fig. 6d), corresponding to metal sulfide (S^2−^) and sulfate (S^6+^), respectively, initially was ~1. Note that the high signal-to-noise ratio of the spectra makes it very difficult to determine whether a double peak due to contribution of different sulfur species actually exists in the case of the low intensity S 2p peak centered at ~161.5 eV. Considering that that the S 2p peak is intrinsically formed by a doublet S 2p_1/2_–S 2p_3/2_ due to spin–orbit coupling with a doublet separation of 1.18 eV, a contribution of the S 2p_1/2_ component at higher binding energies cannot be ruled out. Upon Ar etching for 120 s (i.e., 1.5 nm nominal etch depth), the sulfate content decreased to ~20%. This result suggests the formation of a rather small amount of Hg sulfate, affecting only the outermost pigment surface and being below the detection limit of XRD (Supplementary Fig. 2). The Hg 4f_7/2_ peak remained at ~100.2 eV during Ar etching (Fig. 6e). This value falls between the values reported for Hg^0^ and HgS (i.e., 99.7–99.9 eV and 100.8–101.0 eV)^26^ but has been assigned to weakly bonded elemental mercury^34,35^. According to Qiao et al.^36^, it is associated with Hg^0^ bonded via adsorbed O^−^ species to the cinnabar surface. This finding is in agreement with FESEM observations showing mercury droplets on the altered cinnabar surface (Fig. 6c). Finally, our XPS analyses did not show any Cl in the analyzed samples.

### Photo-induced binder degradation

**Fig. 7:**
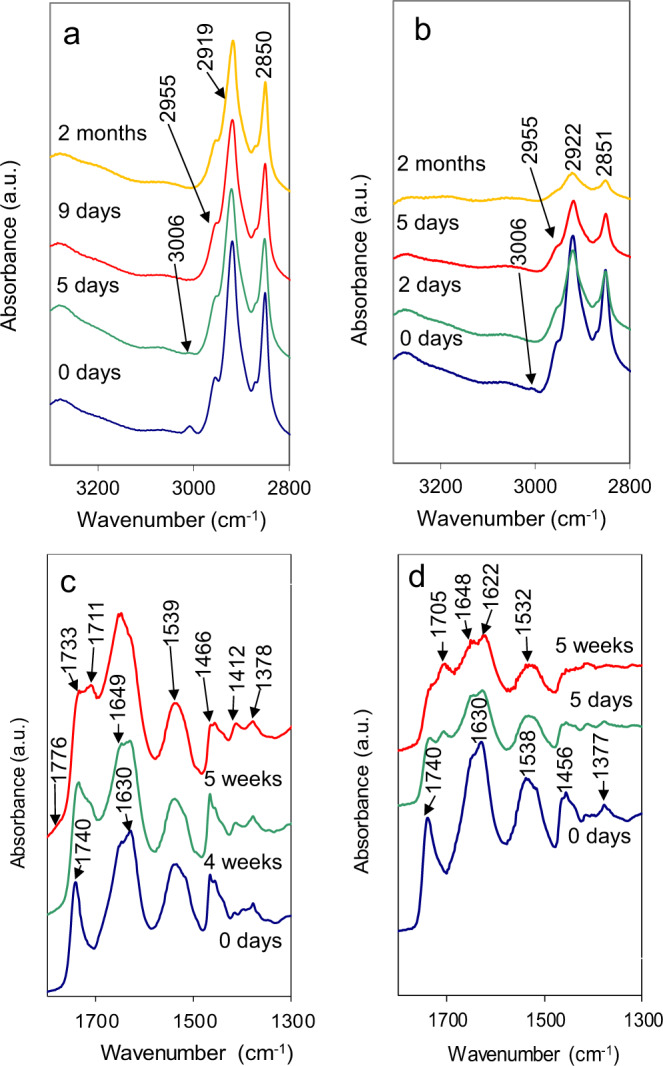
ATR-FTIR spectra of pure egg yolk and cinnabar-based paint. **a**, **c** Egg yolk and **b**, **d** cinnabar-based paint upon UV aging for different periods of time.

According to attenuated total reflectance–Fourier transform infrared spectroscopy (ATR-FTIR) analysis, cinnabar accelerated the photo-induced degradation of the egg yolk-based binder, especially in the case of lipids. The low-intensity band at 3006 cm^−1^, associated with *cis* double-bond stretching vibration of olefinic groups of unsaturated fatty acids^37^, gradually decreased over the first 5 days and completely disappeared after 9 days of UV exposure in the case of pure egg yolk (Fig. 7a). In the presence of cinnabar, this band was only observed in the fresh paint and disappeared after only 2 days of UV exposure (Fig. 7b). It is known that double bonds, apart from ester linkages, are primary sites of reaction in triglycerides, their disappearance being indicative of a very advanced stage of oxidation^37,38^. Further proof for advanced oxidation of the organic binder, especially in the presence of cinnabar, is brought about by a gradual intensity reduction of the CH_2_/CH_3_ bands at 2955, ~2920, and ~2850 cm^−1^ (Fig. 7a, b) as a result of oxidative chain cleavage (-C-H bond peroxidation) yielding volatile oxidation products^39^. The band at 1740 cm^−1^ (Fig. 7c), associated with ester carbonyl functional groups of triglycerides and phospholipids, also gradually decreased in intensity due to a reduction in ester linkages and shifted to 1733 cm^−1^ in pure egg yolk, indicating the presence of aldehyde functional groups or other secondary oxidation products^38,40^. The appearance of a second band at 1711 cm^−1^ after 4-week UV aging is indicative of free fatty acids generated upon hydrolysis of glycerol esters^41^, which can further accelerate the oxidative degradation of lipids^42^. In the presence of cinnabar (Fig. 7d), the intensity of the ester band decreased much faster and a band at 1705 cm^−1^, associated with free fatty acids, was already observed after a 5-day UV exposure. Remarkably, a shoulder at 1776 cm^−1^ was only detected in pure egg yolk, while the presence of cinnabar seemed to have prevented the formation of this feature, which is commonly associated with oxidative polymerization of fatty acids^39^.

Intensity and frequency of characteristic protein–peptide bands (i.e., amide I and II bands) were also affected by UV radiation. In pure egg yolk (Fig. 7c), the decrease in intensity of the amide I band was minor, but the random coil component at 1648 cm^−1^ increased over time at the expense of the β-sheet component at 1630 cm^−1 ^^43^. In the presence of cinnabar (Fig. 7d), the amide I band suffered a rapid decrease in intensity and the component at 1630 cm^−1^ shifted to 1622 cm^−1^, suggesting the presence of aggregated protein as a result of oxidation^44^. The amide II band at 1539 cm^−1^ neither suffered a significant decrease in intensity nor a shift in UV-aged pure egg yolk. In the presence of cinnabar, however, a decrease in intensity together with a small red shift was observed after 5 days of UV aging, which has been previously linked to the formation of stable protein–metal complexes, involving amide groups and Hg^2+^ ions^45^. The δ(CH_2_)n and δ(CH_3_) bands of lipids at 1466 and ~1377 cm^−1 ^^46^, respectively, were little affected in UV-aged pure egg yolk (Fig. 7c) but suffered a significant intensity decrease in the presence of cinnabar, suggesting photo-induced degradation (Fig. 7d).

## Discussion

### Alteration mechanisms of cinnabar pigment

Our results show that cinnabar pigment underwent important mineralogical changes upon UV aging, involving the formation of mercury sulfates (i.e., HgSO_4_·H_2_O and schuetteite (Hg_3_(SO_4_)O_2_)). It might be argued that mercury sulfates would only form under the extreme experimental conditions during UV aging in this study involving UV-C radiation. However, it has to be kept in mind that, due to the relative narrow band gap of HgS, visible light (<620 nm) has a high enough energy to activate the semiconductor. Furthermore, Batrakova et al.^47^ showed that photochemical redox reactions of mercury also take place under UV-A and UV-B radiation (i.e., natural sunlight contains UV-A and part of the UV-B radiation); the intensity and type of radiation having a rate-determining influence. Besides, schuetteite has been found in cinnabar deposits exposed to natural sunlight in numerous locations, including Almadén (Spain), California and Nevada (USA), Bolivia, Moravia (Czech Republic), and Sonora (Mexico)^48^. According to Bailey et al.^19^, this mineral forms through photooxidation of sunlight-exposed cinnabar in the presence of oxygen-bearing surface water. Importantly, the authors acknowledged that HgSO_4_ might be an intermediate phase during schuetteite formation. In any case, sulfate formation is not limited to cinnabar deposits. Radeport et al.^2,17^ acknowledged the possible oxidation of mercury sulfide to sulfate upon cinnabar degradation in the case of a Gothic wall painting from the monastery of Pedralbes (Barcelona, Spain) and detected mercury sulfate in artificially aged cinnabar pellets.

The formation of sulfates upon light exposure is not exclusive to cinnabar. It is long known that CdS-based water colors and oil paint (i.e., CdS is a yellow artists’ pigment) oxidize to CdSO_4_ when exposed to sunlight or UV radiation^20,49^. Both, HgS and CdS, are n-type semiconductors and have extremely low solubility in water^50^. However, they can undergo photo-induced oxidative dissolution upon light irradiation (photocorrosion), resulting in the release of metal ions into solution and the oxidation of sulfide ions^51^. Indeed, photocorrosion of semiconductors has been a widely studied topic as it limits their use for many technical applications such as water splitting or waste water treatment^28^. Hsieh et al.^51^ found that the dissolution of CdS was insignificant in the dark, but increased drastically upon irradiation by light with energy greater than the band gap of CdS (i.e., 2.4 eV corresponding to 512.4 nm). Photoexcitation of semiconductors results in the generation of reactive electron–hole pairs, electrons generated from the conduction band, and holes from the valence band. According to Weng et al.^28^, photo-generated holes are able to oxidize surface sulfide ions (S^2−^) to sulfate (SO_4_^2−^), which would explain the presence of schuetteite and mercury sulfate hydrate on UV-exposed cinnabar pigment grains in our study. They form according to the following overall reaction for the photocorrosion process at high RH (adapted from Meissner et al.^49^, h^+^ = hole):

1 $${{{{{{{\mathrm{HgS}}}}}}}} + 4{{{{{{{\mathrm{h}}}}}}}}^ + + 2{{{{{{{\mathrm{H}}}}}}}}_2{{{{{{{\mathrm{O}}}}}}}} + {{{{{{{\mathrm{O}}}}}}}}_2 \to {{{{{{{\mathrm{Hg}}}}}}}}^{2 + } + {{{{{{{\mathrm{SO}}}}}}}}_4^{2 - } + 4{{{{{{{\mathrm{H}}}}}}}}^ +$$

Considering the potential of redox reactions for mercury sulfate and mercury ions (reactions 2 and 3), it becomes obvious that both fall within the band gap of cinnabar^52^.

2 $$\begin{array}{l}2{{{{{{{\mathrm{HgSO}}}}}}}}_4\left( {{{{{{{{\mathrm{aq}}}}}}}}} \right) + 2{{{{{{{\mathrm{e}}}}}}}}^ - \leftrightarrow {{{{{{{\mathrm{Hg}}}}}}}}_2^{2 + } + 2{{{{{{{\mathrm{SO}}}}}}}}_4^{2 - }\;{{{{{{{\mathrm{is}}}}}}}}\;0.83\,{{{{{{{\mathrm{V}}}}}}}}\;{{{{{{{\mathrm{vs}}}}}}}}{{{{{{{\mathrm{.}}}}}}}}\;{{{{{{{\mathrm{SHE}}}}}}}}\\ \quad ({{{{{{{\mathrm{standard}}}}}}}}\;{{{{{{{\mathrm{hydrogen}}}}}}}}\;{{{{{{{\mathrm{electrode}}}}}}}})\end{array}$$

3 $${{{{{{{\mathrm{Hg}}}}}}}}_2^{2 + } + 2{{{{{{{\mathrm{e}}}}}}}}^ - \leftrightarrow 2{{{{{{{\mathrm{Hg}}}}}}}}\;{{{{{{{\mathrm{is}}}}}}}}\;0.80\,{{{{{{{\mathrm{V}}}}}}}}\;{{{{{{{\mathrm{vs}}}}}}}}{{{{{{{\mathrm{.}}}}}}}}\;{{{{{{{\mathrm{SHE}}}}}}}}$$

This implies that mercury sulfate could be reduced to metallic mercury in sequential reactions via photo-induced electron transfer. Indeed, part of the cinnabar pigment grains suffered darkening upon UV exposure, and a few nano-sized droplets, presumably metallic mercury, were observed on the pigment surface. However, massive amounts of yellow schuetteite (with a 3/1 Hg/S ratio), which covered large parts of the UV-aged pigment grains acted as a sink for mercury ions and limited the formation of metallic mercury. Schuetteite formation appears to be facilitated by the extreme conditions upon UV aging at high RH. Under more moderate aging conditions, photocorrosion will proceed at a slower rate and mercury sulfate formation will be more restricted, as observed in the case of cinnabar paint where pigment grains were to some extent protected from photocorrosion by the organic binder (see below). Our experimental results also showed that part of the sulfur released upon cinnabar decomposition was consumed during sulfation of accessory minerals. Sulfation could also affect other mineral phases in direct contact with the cinnabar pigment, such as calcium carbonate-based painting grounds in mural or oil paintings, and should be considered in the analysis of painting materials.

### Alteration mechanisms of cinnabar-based tempera paint

In the case of tempera paint, UV aging at high RH led to significant binder degradation and darkening. It might be argued that the important conformational changes undergone by the organic binder would only occur under the highly oxidative conditions during UV testing. However, similar modifications have been reported for naturally aged (i.e., long-term light exposure under ambient museum conditions) paints containing egg yolk^39^. Photooxidation of organics at high RH involves the generation of oxidants and free radicals, either via UV-photolysis of oxygen and water molecules or, in the case of lipids, by a series of auto-catalyzed reactions leading to the formation of free peroxy, alkoxy, and lipid radicals. The latter radicals also facilitate protein degradation^53^.

Comparison of ATR-FTIR data for pure egg yolk and cinnabar-based tempera paint revealed a drastic acceleration of the oxidative degradation of lipids and proteins in the presence of the cinnabar pigment. The findings of our study are in agreement with previously published results revealing an accelerating effect of cinnabar on photo-induced aging (i.e., cross-linking, hydrolysis, and oxidation) of various natural organic binders such as drying oils, egg albumin, egg yolk, and casein^54–56^. However, paint degradation was not limited to the organic binder and also affected the inorganic pigment, which underwent severe darkening. In contrast to the pure pigment, formation of mercury sulfate was limited and only detected in the outermost, nanometer-thick surface layer of UV-aged paint according to XPS. We hypothesize that the presence of the organic binder limited pigment degradation and the concomitant formation of mercury sulfate phases by: (a) forming a protective film, which acted as physical barrier and a photochemical filter for UV radiation (Supplementary Fig. 4) and hindered the direct access of oxidants and radicals generated during UV aging; and (b) via a competing depletion of photo-generated holes by the organic binder in direct contact with the semiconductor, which consequently led to the oxidation of lipids and proteins. The role of semiconductors in the photooxidation of organic molecules has long been recognized and their use has been proposed for various technical applications such as waste water treatment involving the decomposition of organics^57^. Our hypothesis is supported by the fact that a similar photocatalytic activity has been recognized for other semiconductor pigments including minium (Pb_3_O_4_) and titanium white (TiO_2_), which are reported to facilitate photooxidation of oil binders at the pigment surface^58,59^. Further proof for a competing depletion of holes is brought about by Harada et al.^60^, who studied the photocatalytic decomposition of lactic acid by CdS. The authors state that 99.6% of photo-generated holes were consumed by the decomposition of the organic acid, a rapid reaction which drastically limited the photooxidation of CdS.

According to our FESEM and XPS results the UV-aged paint surface was covered by elemental mercury, likely bonded via O^-^ species. It seems reasonable to assume that metallic mercury formed in sequential reactions (Eqs. 1–3) via photo-induced electron transfer. In the case of the UV-aged paint, no evidence for the formation of schuetteite was obtained, which competes/acts as a sink for mercury ions and consequently restricted the formation of Hg^0^. In addition, organics likely provided active sites, facilitating the sorption of a sufficient amount of Hg^0^ to cause a visible darkening of the paint surface. Indeed, previous studies on the Hg^0^ adsorption capacity of activated carbon have shown that different oxygen functional groups including carbonyl and carboxyl groups can provide active sites for chemisorption of Hg^0 ^^61^. Chemisorption via functional groups would also explain why the nano- and micrometer-sized mercury droplets on the degraded paint surface withstood the high vacuum and impact of the electron beam during FESEM analysis, unlike the droplets observed on the UV-aged pigment, which readily evaporated.

In this study, unambiguous direct evidence obtained for the presence of metallic mercury droplets on the UV-exposed tempera paint surface confirms the widely accepted hypothesis that metallic mercury is responsible for cinnabar darkening. Our experimental results furthermore demonstrate that cinnabar darkening was driven by a process involving the oxidation of mercury sulfide to sulfate and the subsequent reduction to metallic mercury via photo-induced electron transfer, which can be considered an alternative to the often reported Cl-mediated pathway.

## Methods

### Pigment and binder

Natural Chinese cinnabar pigment (Ref. No. 10627, Kremer Pigmente GmbH & Co. KG, Germany) from the Hunan district with a grain size between 15–90 μm was used in this study^18^. Tempera paints were prepared with fresh egg yolk. Egg yolk has a solid content of ~50 wt% and contains 31.8–35.5 wt% lipids, 15.7–16.6 wt% proteins, 1.1 wt% ash, and 0.2–1.0 wt% carbohydrates^62^. The lipid fraction is made up of ~30 wt% phospholipids and ~60 wt% triglycerides, the latter containing about one-third saturated fatty acids (i.e., stearic and palmitic acids) and about two-thirds unsaturated acids (i.e., oleic and linoleic acid)^63^. Its elemental composition, besides C, H, and O, includes K, P, Ca, and Mg in concentrations ≤1 wt%, and S, Fe, and Na in concentrations ≤0.1 wt%^62^.

### Sample preparation

Cinnabar pigment (1 g) was mixed with 3 mL Milli-Q water and uniformly applied with a spatula on circular glass slides (Ø = 33 mm). Cinnabar-based egg tempera paint was prepared based on organoleptic parameters according to traditional methods used by medieval painters^64^ and applied in several layers on glass slides by brush. Additionally, pure egg yolk was applied to glass slides in an identical manner. Samples were dried under laboratory conditions (~20 °C and ~40% RH). For details see Elert and Cardell^18^. The final paint layer was 0.15 ± 0.03 mm thick and had a binder content of 14 wt% (dry weight). The size of the mock-ups was 23 × 20 mm.

### UV aging

Pigments and paint mock-ups were exposed to UV radiation emitted by a small tubular Pen-Ray Mercury Lamp (No. 90-0012-01, Utra-Violet Products Ltd, UK). The lamp emits a spectrum from 185–436 nm with the primary energy at 254 nm (2800 μW/m^2^ UV-C irradiance at ~2.5 cm). Mercury lamps are frequently used for ozone production, since wavelengths <240 nm cause photolysis of oxygen molecules generating two oxygen atoms, which combine with oxygen molecules to form ozone. During testing, pigments and paint mock-ups were placed in a glass test chamber (9 L) at 21 ± 2 °C and 80 ± 3% RH. RH was controlled by placing a 150 mL glass beaker with Milli-Q water inside the vessel. Under the high RH conditions during UV aging the generation of other reaction products, apart from ozone, are expected, including free radicals and oxidants such as hydroxyl (OH^•^), superoxide (O_2_^•^^−^), singlet oxygen (^1^O_2_), and hydrogen peroxide (H_2_O_2_). All of these are highly reactive and readily attack organic and inorganic substrates^65,66^. Daylight was blocked using aluminum foil, which was attached with double sided tape to the outside of the test chamber. The overall UV exposure duration was 2 months.

### Analytical techniques

#### Photographic imaging and optical microscopy

Digital imaging (Canon EOS D30, Japan) and optical microscopy in reflection mode (SMZ 1000, Nikon, Japan) were used to study the textural and chromatic features of control and aged pigments and paint mock-ups before and after UV aging.

#### Spectrophotometry

A portable spectrophotometer (CM-700d, Minolta, Japan) was used to determine color changes of mock-ups before and after exposure to UV radiation. Equipment settings: illuminant D65, 10° observer, and Ø 6 mm measurement area. Data were expressed in the CIE L*a*b* color space (i.e., L* is luminosity or lightness which varies from black with a value of 0 to white with a value of 100; a* varies from +a* (red) to −a* (green) and b* from +b* (yellow) to −b* (blue). Color change was calculated using the following formula: ∆E* = (∆L*^2^ + ∆a*^2^ + ∆b*^2^)^1/2^. Note that calculations are only based on SCI (specular component included) values because the difference between SCI and SCE (specular component excluded) measurements was negligible. Average values are based on a minimum of 10 measurements per sample. Note that a total color differences of ΔE* = ≤3 is generally not perceptible to the human eye^67^.

#### Micro X-ray fluorescence (μ-XRF)

The pigment and paint chemical composition was determined with μ-XRF mapping using a M4 Tornado (Bruker, USA), operating at 50 kV and 600 μA. Analyzed areas were 8.9 mm^2^ (×10) and 1.3 mm^2^ (×100), respectively.

#### Ultraviolet-visible-near infrared spectroscopy (UV-Vis-NIR)

Absorption of UV and visible light of egg yolk has been determined using a Varian Cary 5E Spectrophotometer (SpectraLab Scientific Inc., Canada).

#### Field emission scanning electron microscopy

Morphological features and chemical composition of carbon coated pigments and mock-ups were studied using an Auriga (Carl Zeiss, Germany) coupled to an INCA-200 EDS system. Working conditions: 10^−6^ vacuum, 3 kV beam accelerating voltage in secondary electron imaging mode, 10 kV in backscattering imaging mode, and 20 kV for microanalysis.

#### X-ray diffraction

The mineralogical composition of cinnabar pigments and paint mock-ups before and after UV aging was determined using XRD (X’Pert PRO PANalytical B.V., The Netherlands). Equipment settings: Cu-Kα radiation, Ni filter, 45 kV voltage, 40 mA intensity, 3°–60° 2*θ* exploration range, and 0.05° 2*θ* /s goniometer speed. Identification of minerals and the determination of the peak intensity and crystallite size (Scherrer equation) were carried out using the Xpowder software^68^.

#### Attenuated total reflectance–Fourier transform infrared spectroscopy

Conformational changes of pure egg yolk and paint mock-ups upon UV aging were studied using ATR-FTIR spectroscopy (Jasco 6200, JASCO Analytical Instruments, Japan). Small paint chips were directly pressed against the ATR diamond crystal window. Pure binder was analyzed as a dry powder. Infrared spectra were recorded at 2 cm^−1^ resolution over 75 scans from 400 to 4000 cm^−1^.

#### X-ray photoelectron spectroscopy

Quantitative elemental composition and oxidation state of Hg and S of unaltered and UV-aged cinnabar pigments and mock-ups were determined with an Axis Ultra-DLD (Kratos Analytical Ltd., UK), using monochromatic Al Kα radiation. Equipment settings: Survey spectra at 75 W X-ray source power and 160 eV pass energy, and high-resolution spectra of C, Hg, S, and Cl at 225 W source power and 20 eV pass energy, 1.33 × 10^−8^ Pa vacuum. The analyzed area was ~300 × 700 µm in size. Qualitative analysis and calculation of abundance percentage of sulfur and mercury species were performed using the CasaXPS software (Casa Software Ltd., UK). Hg and S binding energies were determined using the C 1s transition at 284.6 eV as reference. The positions of the multiple doublets S 2p_3/2_–S 2p_1/2_ were determined using a binding energy difference constrained to 1.2 eV and an intensity ratio of 2:1. Ar ion beam etching (4 keV energy, 10 mA emission current) was used to obtain depth profiles (3.5 × 3.5 mm raster size). The nominal etch depth was determined using a calibration standard (i.e., 60 nm Ta oxide layer on a Ta foil), which was etched under identical conditions as the samples to be analyzed. The calculated nominal etch rate was 0.75 nm/min. Note that prolonged ion beam etching (>10 min) can cause preferential Hg removal^69^, leading to surface alteration visible under FESEM as we have previously observed in the case of naturally aged cinnabar mock-ups^18^. To avoid any experimentally induced changes, ion beam etching was limited to 2 min.

## Supplementary information

Supplementary Information

## Data Availability

The authors declare that all data supporting the findings of this study are available within the paper and its Supplementary Information file. Additional data related to this paper may be requested from the authors. Correspondence and requests for materials should be addressed to K.E. (kelert@ugr.es).
