# Supplementary material for: Direct evidence for metallic mercury causing photo-induced darkening of red cinnabar tempera paints
Source: Commun Chem. 2021 Dec 10;4:174. doi: 10.1038/s42004-021-00610-2 (PMC9814095; doi:10.1038/s42004-021-00610-2)
Supplement: Supplementary file 1 — Supplementary Information [file 42004_2021_610_MOESM1_ESM.pdf]

Supplementary Information for “Direct evidence for metallic mercury causing photo-induced darkening of red cinnabar tempera paints”

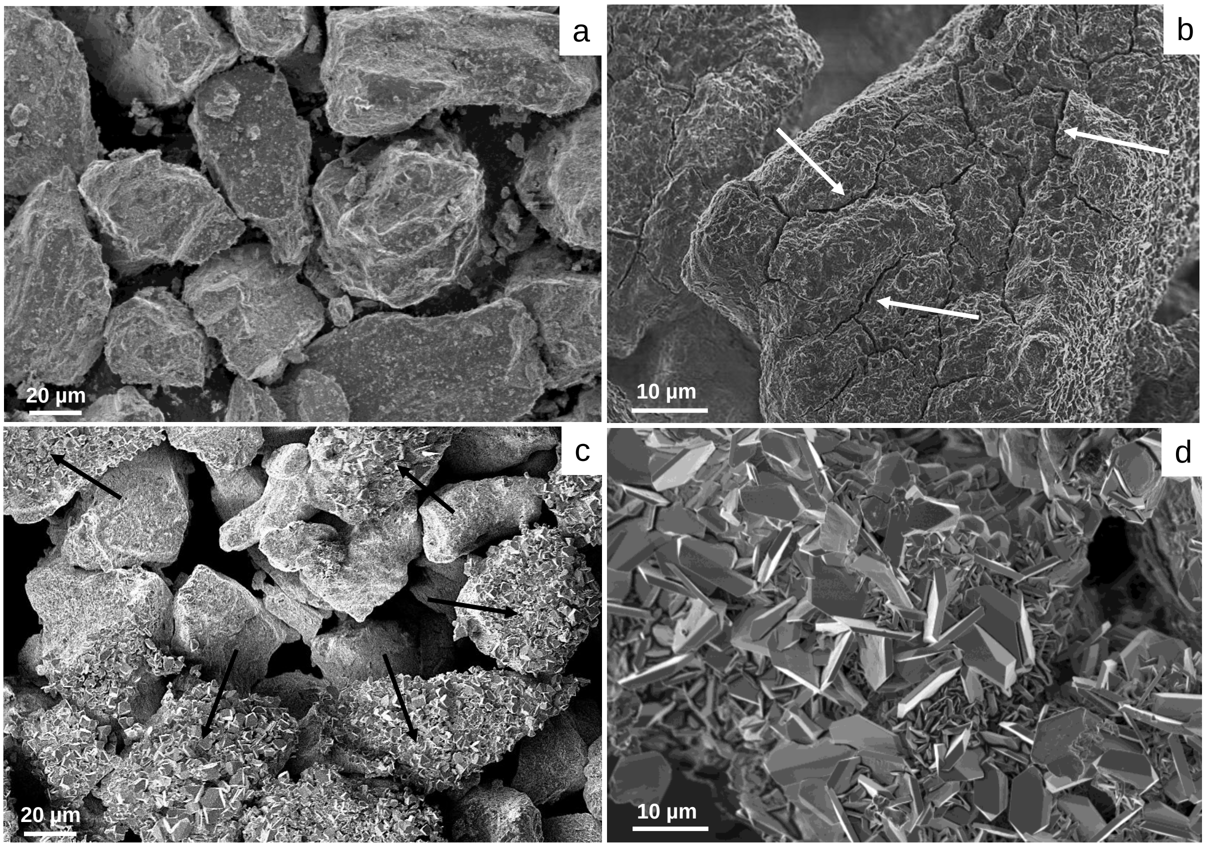

Supplentary Fig.1. FESEM images of unaltered and UV-aged cinnabar pigment. a) unaltered cinnabar pigment grains, b) cinnabar pigment after 2 months of UV aging showing grains with extensive crack formation (arrows), c) UV-aged grains partially covered by schuetteite (arrows), and d) detail of the UV-exposed pigment surface showing $HgSO_4 \cdot H_2O$ and schuetteite.

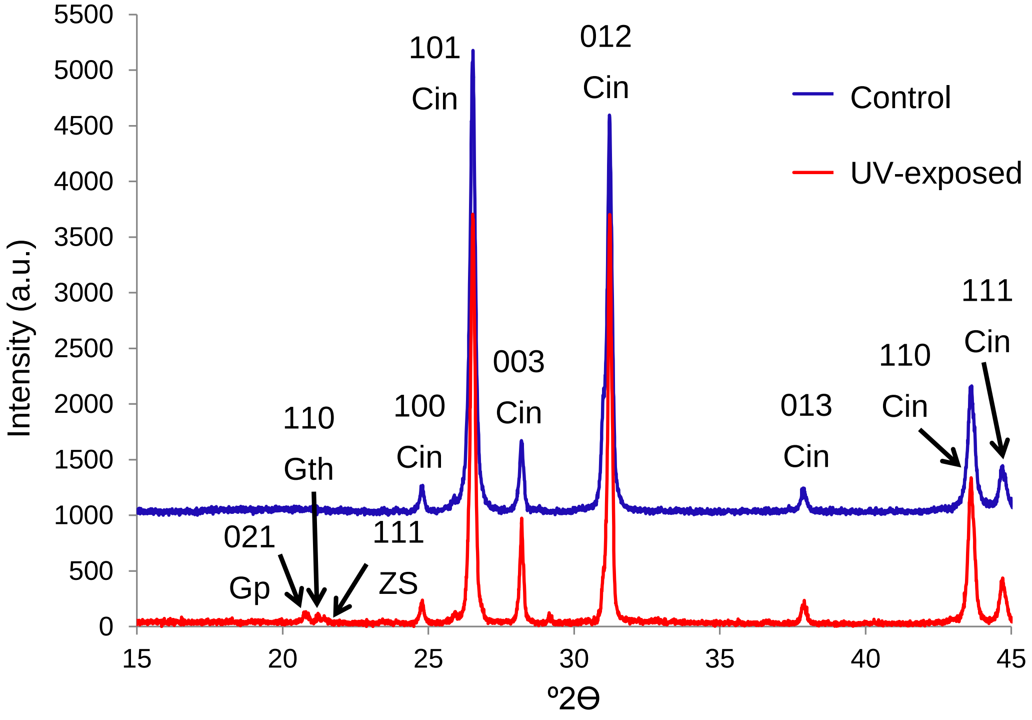

Supplementary Fig. 2. XRD patterns of unaltered and UV-exposed cinnabar paint. Gp = gypsum. Gth = goethite, ZS = zinc sulfate, and Cin = cinnabar (Mineral abbreviation, except zinc sulfate, according to Whitney and Evans (*Am. Mineral.* **95,** 185-187 (2010)).

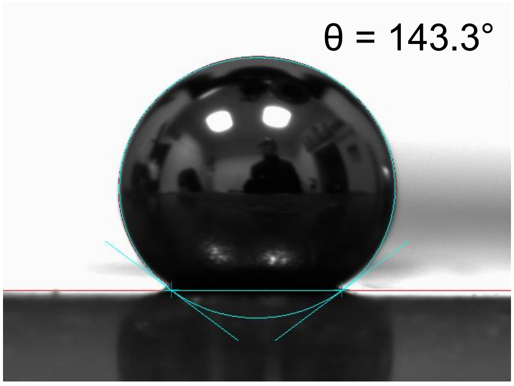

Supplementary Fig. 3. Contact angle measurement of a 3 µl mercury droplet on a polished cinnabar surface using the sessile drop method (OCA 15EC, DataPhysics Instruments, Germany).

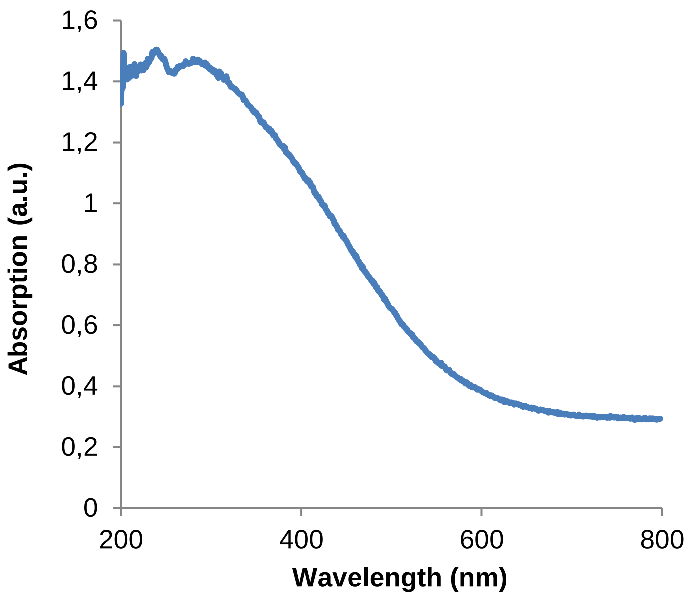

Supplementary Fig. 4. UV-Vis spectrum of egg yolk revealing important absorption for $\lambda \leq 550$ nm.

Supplementary Table 1. Normalized elemental concentrations (wt%) based on µ-XRF mapping.

Sample	Hg	S	Mg	P	Ca	K	Na	Fe	Ba
Pigment	84.26	11.71	0.26	0	1.91	0.02	0	0.28	1.56
Paint	82.96	11.59	0.04	1.49	1.35	0.53	0.37	0.12	1.55

Supplementary Table 2. Crystallite size (nm) for different *hkl* Bragg peaks of cinnabar pigment before (control) and after UV aging (UV-exposed)

Sample	102	110	111	014	201	113
Control	51	37	35	38	33	33
UV-exposed	46	34	31	34	31	30
